# Impact of a magnetic resonance imaging-guided treat-to-target strategy on disease activity and progression in patients with rheumatoid arthritis (the IMAGINE-RA trial): study protocol for a randomized controlled trial

**DOI:** 10.1186/s13063-015-0693-2

**Published:** 2015-04-21

**Authors:** Signe Møller-Bisgaard, Kim Hørslev-Petersen, Bo Jannik Ejbjerg, Mikael Boesen, Merete Lund Hetland, Robin Christensen, Jakob Møller, Niels Steen Krogh, Kristian Stengaard-Pedersen, Mikkel Østergaard

**Affiliations:** Department of Rheumatology, Copenhagen University Hospital, Slagelse, Denmark; King Christian 10th Hospital for Rheumatic Diseases, Graasten and Institute of Regional Health research, University of Southern Denmark, Odense, Denmark; Department of Radiology and the imaging unit, The Parker institute, Copenhagen University Hospital, Bispebjerg-Frederiksberg, Copenhagen F, Denmark; DANBIO registry and Copenhagen Centre for Arthritis Research, Centre for Rheumatology and Spine Diseases, Copenhagen University Hospital, Glostrup, Denmark; Musculoskeletal Statistics Unit, The Parker Institute, Department of Rheumatology, Copenhagen University Hospital, Bispebjerg and Frederiksberg, Copenhagen F, Denmark; Department of radiology, Copenhagen University Hospital, Herlev, Denmark; ZiteLab ApS, Copenhagen, Denmark; Department of Rheumatology, Aarhus University Hospital, Aarhus, Denmark

**Keywords:** Rheumatoid arthritis, Magnetic resonance imaging, Bone marrow edema, DAS28 remission, Erosive progression

## Abstract

**Background:**

Rheumatoid arthritis (RA) is a chronic, progressive joint disease, which frequently leads to irreversible joint deformity and severe functional impairment. Although patients are treated according to existing guidelines and reach clinical remission, erosive progression still occurs. This demonstrates that additional methods for prognostication and monitoring of the disease activity are needed. Bone marrow edema (BME) detected by magnetic resonance imaging (MRI) has proved to be an independent predictor of subsequent radiographic progression. Guiding the treatment based on the presence/absence of BME may therefore be clinically beneficial. We present the design of a randomized controlled trial (RCT) aiming to evaluate whether an MRI-guided treatment strategy compared to a conventional treatment strategy in anti-CCP-positive erosive RA is better to prevent progression of erosive joint damage and increase the remission rate in patients with low disease activity or clinical remission.

**Methods/design:**

The study is a non-blinded, multicenter, 2-year RCT with a parallel group design. Two hundred anti-CCP-positive, erosive RA patients characterized by low disease activity or remission, no clinically swollen joints and treatment with synthetic disease-modifying antirheumatic drugs (DMARDs) will be included. Patients will be randomized to either a treatment strategy based on conventional laboratory and clinical examinations (control group) or a treatment strategy based on conventional laboratory and clinical examinations as well as MRI (intervention group). Treatment is intensified according to a predefined treatment algorithm in case of inflammation defined as a disease activity score (DAS28) >3.2 and at least one clinically swollen joint (control and intervention groups) and/or MRI-detected BME (intervention group only). The primary outcome measures are DAS28 remission (DAS28 < 2.6) and radiographic progression (Sharp/vdHeijde score).

**Discussion:**

The perspectives, strengths and weaknesses of this study are discussed.

This study has been approved by The Regional Scientific Ethical Committees for Southern Denmark, S-20110109. Dissemination will occur through presentations and publication in international peer-reviewed journals.

**Trial registration:**

The study is registered in http://www.ClinicalTrials.gov identifier: NCT01656278 (5 July 2012)

**Electronic supplementary material:**

The online version of this article (doi:10.1186/s13063-015-0693-2) contains supplementary material, which is available to authorized users.

## Background

Rheumatoid arthritis (RA) is a chronic inflammatory joint disease, which primarily affects the small joints of the hands and feet, with a prevalence of 0.5-1.0% in the adult population [1]. Patients typically experience joint pain, disability and loss of quality of life, and they are at risk of developing progressive joint damage, which eventually leads to irreversible joint deformity and severe functional impairment [2]. The long-term prognosis of RA is poor. After 20 years of disease, 80% of the patients will have evidence of disability, and 19% are severely disabled [3]. Approximately 50% of the patients will have stopped working 10 years after disease onset [4].

Erosive joint damage occurs early in the disease course and precedes subsequent progressive joint damage and functional limitation [5,6]. Other known prognostic factors of radiographic damage are existing radiographic damage, shared epitope alleles, rheumatoid factor (RF) and/or anti-citrullinated peptide (anti-CCP) antibody positivity and biochemical signs of inflammation [increased erythrocyte sedimentation rate (ESR) and C-reactive protein (CRP)] at disease onset [7]. However, even though these prognostic factors are all associated with poor radiographic outcome on a group level, at the individual patient level they cannot distinguish which patients will progress or not. MRI bone marrow edema, which reflects inflammatory infiltrates in the bone marrow (osteitis) [8,9], has proved to be a strong independent predictor of radiographic progression in patients with early RA [10-12] and may therefore have significant prognostic value at an individual level.

The modern treatment strategy involves early and aggressive treatment with frequent clinical follow-up aiming at reaching a target of clinical remission in patients with early RA and at least a state of low disease activity in patients with longstanding RA [13]. This treat-to-target strategy has been shown to slow the destructive progression and prevent functional loss [14,15]. However, it has been demonstrated that 20-30% of patients who reach the treatment target of clinical remission still show progressive erosive joint damage, no matter what remission criteria are used [16,17].

Thus, the clinical and laboratory methods currently used in routine clinical practice for assessments of disease activity are not sufficiently sensitive. This illustrates and emphasizes that new more sensitive methods for monitoring and prognostication are highly needed to ensure that the treatment can be optimally adjusted in order to achieve the best possible outcome.

MRI depicts the pathological changes in all tissues involved in RA and shows greater sensitivity in detecting inflammatory and destructive changes than both clinical examination and x-ray [18,19]. This superior sensitivity of MRI compared to conventional clinical examinations and radiographs, combined with the knowledge that MRI detected BME is a strong predictor of subsequent radiographic progression, has generated the hypothesis that adding MRI to the conventional clinical and laboratory examinations and intensifying treatment in the presence of subclinical MRI BME will reduce radiographic erosive progression and improve the patient’s functional level.

The aim of the present study is in an RCT to evaluate whether an imaging-based treat-to-target treatment strategy using MRI and DAS 28 (Disease Activity Score involving 28 joints) can prevent progression of erosive joint damage and increase the remission rate in patients with RA compared to a treat-to-target treatment strategy guided only by DAS 28. To our knowledge, this is the first study using MRI as a guide for treatment intensification in RA patients.

## Methods/design

**Figure 1:**
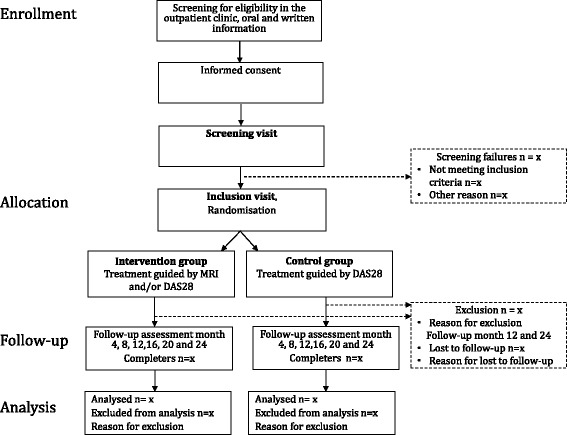
Flow chart. Flow chart through the phases of the IMAGINE-RA study from enrollment to analysis.

The study is conducted in Denmark as a multicenter, non-blinded, 2-year, RCT with a parallel group design. A diagram of the phases (enrollment, allocation, follow-up, analysis) through the trial is shown in Figure 1.

### Participants

Participants will be recruited to the study from the rheumatology outpatient clinics at ten Danish Hospitals: Copenhagen University hospitals at Glostrup, Slagelse, Gentofte, Bispebjerg-Frederiksberg and Køge; Aarhus University hospitals at Aarhus and Silkeborg; university hospitals of Southern Denmark at Odense and Graasten; Ålborg University hospital at Hjørring. The rheumatologist will ask eligible patients at a routine visit whether they would consider participating in the study. Oral and written information will be provided. If the patient agrees to participate, declaration of informed consent is signed. Patients will not be recruited through advertising. A screening visit is carried out to ensure that patients meet the criteria for participation.

Inclusion criteria are: RA patients diagnosed according to the ACR (American College of Rheumatology)/EULAR (European League Against Rheumatism) 2010 criteria [20], ≥18 years of age, low disease activity/remission (defined as DAS28-CRP <3.2 and no clinically swollen joints assessed clinically by the treating rheumatologist), bone erosion on radiography (described by the local radiologist), anti-CCP positivity (above the upper normal limit according to the local laboratory range), and treatment with DMARDs in mono- or combination therapy (methotrexate, sulfasalazine, hydroxychloroquine, leflunomide) in a stable dose during the previous 6 weeks. Exclusion criteria are: former treatment with biologic agents, known intolerance to methotrexate (patient should tolerate at least 7.5 mg/week), intramuscular, intraarticular or intravenous glucocorticoid administration <6 weeks prior to inclusion, oral glucocorticoid administration >5 mg/day, changes in oral glucocorticoid dose <3 months prior to inclusion, liver enzymes >2× the upper limit of normal range at screening, current and/or imminent wish to become pregnant, contraindications for TNF-alpha-inhibiting treatment, contraindications for MRI, known alcohol/drug abuse, and a physical or mental state that impedes the ability to give informed consent and show compliance with the study program. Non-protocoled use of MRI or ultrasonography to visualize joint inflammation is not allowed during screening or study periods.

### Interventions

**Figure 2:**
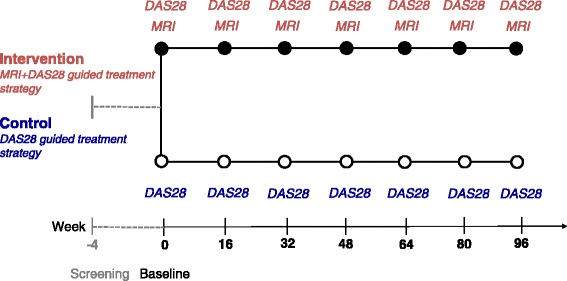
Intervention. The participants will be randomized 1:1 into either a treatment strategy based on DAS28 (control group) or a treatment strategy based on DAS28 and MRI (intervention group). Disease activity will be monitored systematically every 4 months by the CRP-based disease activity score (DAS28-CRP) (control and intervention groups) and presence of MRI BME (intervention group only).

Participants are randomized into one of two groups: (A) a treatment strategy based on DAS28 (control group) or (B) treatment strategy based on DAS28 and MRI (intervention group). Disease activity will be monitored systematically every 4 months by the CRP-based DAS28 (DAS28-CRP) [21,22] (intervention and control groups) and by DAS28-CRP and MRI of the dominant hand and wrist (intervention group only) (Figure 2).

### Control group

**Figure 3:**
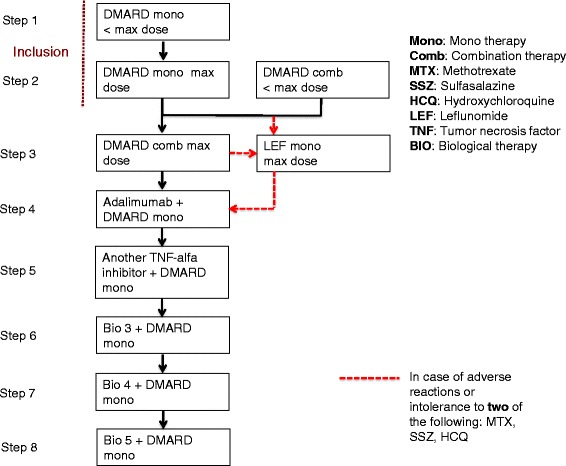
Treatment algorithm. Step 1: DMARD monotherapy at less than the maximum tolerated dose (maximum dose: up to MTX 25 mg/week, SSZ 3 g/day, HCQ 400 mg/day or LEF 20 mg/day). Step 2: DMARD monotherapy at the maximum tolerated dose (maximum dose: up to MTX 25 mg/week, SSZ 3 g/day, HCQ 400 mg/day or LEF 20 mg/day. Minimum dose: min. MTX 7.5 mg/week, SSZ 1 g/day, HCQ 200 mg/day, LEF 10 mg/day). Step 2a: DMARD combination therapy at less that the maximum tolerated doses (maximum dose up to MTX 25 mg/week, SSZ 2 g/day and HCQ 200 mg/day). Step 3: DMARD combination therapy at the maximum tolerated doses (maximum dose up to MTX 25 mg/week, SSZ 3 g/day and HCQ 400 mg/day). Step 3a: LEF (in case of adverse reactions or intolerance to two of the following: MTX, SSZ, HCQ in three-drug therapy, change to monotherapy LEF 20 mg/day without prior loading dose). Step 4: MTX or LEF or SSZ (prioritized sequence) at the maximum tolerated dose + adalimumab* Step 5: MTX or LEF or SSZ (prioritized sequence) at the maximum tolerated dose + other TNF-alpha inhibitor* (selected by the attending rheumatologist). Steps 6, 7 and 8: MTX or LEF or SSZ (prioritized sequence) at the maximum tolerated dose + biological treatment* (preferably non-TNF-alpha inhibitor). *Biological treatment is administered in combination with DMARD monotherapy treatment in the following prioritized order: MTX, LEF, SSZ. If the patient develops adverse reactions to the supplemented DMARD monotherapy treatment and is unable to tolerate the minimum dose, a switch must be made to another DMARD in the mentioned prioritized order.

If the patients at a scheduled visit shows *unsatisfactory inflammatory activity*, which is defined as the presence of at least one clinically swollen joint (assessed by the rheumatologist) and DAS28-CRP >3.2, the treatment will be intensified according to the predefined treatment algorithm (Figure 3).

### Intervention group

The intervention group will, in addition to treatment intensification based on the DAS28-CRP, have their treatment intensified in case of MRI BME. Thus, treatment is intensified according to the predefined treatment algorithm if the patient exhibits *unsatisfactory inflammatory activity,* which is defined as the presence of at least one clinically swollen joint (assessed by the rheumatologist) and DAS28-CRP >3.2 and/or MRI-detected BME.

### Treatment algorithm

At the time of inclusion, the patient will receive DMARD monotherapy treatment in less than the maximum or maximum tolerated dose (step 1 or 2) or combination treatment (2a) in the form of two- or three-drug therapy (Figure 3). If the patient is receiving three-drug therapy, at least one of the preparations must be administered at less than the “maximum inclusion dose,” which is defined as methotrexate (MTX) 25 mg/week (or the maximum tolerated dose if 25 mg/week is not tolerated), sulphasalazine (SSZ) 2 g/day (or the maximum tolerated dose if 2 g/day is not tolerated) and hydroxychloroquine (HCQ) 200 mg/day (or the maximum tolerated dose if 200 mg/day is not tolerated). If the patient exhibits *unsatisfactory inflammatory activity* at step 1, the dose of the DMARD is escalated within 4 weeks to the maximum tolerated dose (step 2). If the patient exhibits *unsatisfactory inflammatory activity* at treatment step 2, the patient moves one step up the treatment ladder to DMARD combination therapy (MTX, SSZ, HCQ) at the maximum tolerated doses (step 3). If less than two of these are tolerated at the minimum dose, treatment with leflunomide (LEF) monotherapy is started before another step up the treatment ladder. From step 3 to 4 the patient switches treatment to tumor necrosis factor (TNF)-alpha inhibitor treatment, which at step 4 will be adalimumab treatment combined with DMARD monotherapy in the form of MTX, LEF or SSZ (in prioritized sequence) at the maximum tolerated dose. From step 4 to 5, the patients switch to a different TNF-alpha inhibitor in accordance with the local guidelines. Supplementary treatment with MTX, LEF or SSZ monotherapy (in prioritized sequence) at the maximum tolerated dose is continued. At treatment steps 6, 7 and 8, the patient is switched to a different biological treatment (preferably with another mode of action than TNF-alpha inhibitors) combined with DMARD monotherapy as mentioned above in accordance with the local guidelines. If the patient does not at any time during the study period exhibit *unsatisfactory inflammatory activity*, the inclusion treatment will be maintained throughout the study period.

In both the control and intervention group, intraarticular glucocorticoid treatment *must* be administered at the ordinary 4-monthly visits (maximum 4 ml) in clinically swollen joints and *may* be administered in the intervening 4-month period (further maximum of 4 ml glucocorticoids per 4-month period). However, glucocorticoid administration should be *avoided* <6 weeks before the scheduled ordinary visit. NSAID (non-steroid anti-inflammatory drug) treatment is only permitted in stable doses during the study period.

Stepping up the treatment ladder can only occur at the ordinary 4-month study visit in the case of *unsatisfactory inflammatory activity* as described above. An exception is if the patient exhibits *“unacceptably high disease activity,”* which is defined as DAS28-CRP >5.1 and ≥1 clinically swollen joint, with the findings not possibly being explained by a different disease. Then, if the patient has not changed treatment at the recent study visit, and the patient already has received the permitted dose of intraarticular glucocorticoids according to the protocol, the patient is allowed to move one step up the treatment ladder, and the following visit will be 4 months after the treatment changes.

### Procedures

During the 2-year study period, the patients will have seven visits in the outpatient clinic with 4-month intervals (see Figure 2). The time from the screening visit to inclusion should not exceed 4 weeks. An overview of visits and corresponding assessments is shown in Table 1. If the patient is excluded/withdrawn from the study, the patient is asked to attend follow-up visits at the time points for visits 4 and 7 (years 1 and 2) having the related procedures performed.

**Table 1:** **Summary of measures to be collected**

**Variable**								
**Visit number**	**0/Screening**	**1/baseline**	**2**	**3**	**4**	**5**	**6**	**7**
**Week**	**−4-0**	**0**	**16**	**32**	**48**	**64**	**80**	**96**
**Demographic data**								
Age – years	A	-	-	-	-	-	-	-
Female sex – no. (%)	A	-	-	-	-	-	-	-
Disease duration	A	-	-	-	-	-	-	-
Duration of RA symptoms	A	-	-	-	-	-	-	-
Smoking status	A	-	-	-	-	-	-	-
Marital status	A	-	-	-	-	-	-	-
Educational and employment status	A	-	-	-	-	-	-	-
***Medical history***								
Former and current arthritis treatment	A	-	-	-	-	-	-	-
Concomitant medication	A	A	-	-	A	-	-	A
***Physical examination***								
40 joint assessment* (TJC/SJC)	A	A	A	A	A	A	A	A
General physical examination**	A	A	-	-	A	-	-	A
Height – cm	A	A	-	-	A	-	-	A
Weight – kg	A	A	-	-	A	-	-	A
Blood pressure and pulse	A	A	A	A	A	A	A	A
Electrocardiogram	A	-	-	-	-	-	-	-
***Laboratory assessment***								
Routine blood tests***	A	A	A	A	A	A	A	A
Anti-CCP and IgM-RF	A	-	-	-	A	-	-	A
Biomarker collection (blood and urine) for bio-bank	-	A	A	A	A	A	A	A
***Patient reported outcomes***								
Patient: VAS pain, VAS global, VAS fatigue – range 0-100	A	A	A	A	A	A	A	A
Morning stiffness - min	A	A	A	A	A	A	A	A
HAQ	A	A	A	A	A	A	A	A
SF-36	-	A	-	-	A	-	-	A
EQ-5D	-	A	-	-	A	-	-	A
***Physician’s global assessment of disease activity (VAS) 0-100***	A	A	A	A	A	A	A	A
DAS28 (CRP) score****	A	A	A	A	A	A	A	A
**Imaging**								
X-ray hands and feet	-	A	-	-	A	-	-	A
*Controls* MRI 2nd to 5th MCP and wrist	-	A	-	-	A	-	-	A
Dynamic MRI*****	-	A	-	-	A	-	-	A
*Intervention* MRI2nd to 5th MCP and wrist	-	A	A	A	A	A	A	A
Dynamic MRI*****	-	A	A	A	A	A	A	A

A = Assessed; − = not assessed; TJC = tender joint count; SJC = swollen joint count; anti-CCP = anti-citrullinated peptide; IgM-RF = IgM-rheumatoid factor; VAS = visual analog scale; HAQ = health assessment questionnaire; SF-36 = short form (36) health survey; EQ-5D = EuroQol; DAS28 = disease activity score based on assessment of 28 joints; MRI = magnetic resonance imaging; MCP = metacarpophalangeal joints.

*Right and left shoulder joints, elbow joints, wrists, MCP joints, PIP joints, 1st IP joint, knee joints, ankle joints and MTP joints.

**Inspection of the cavum oris, skin, assessment of lymph node status, heart and lung stethoscopy, examination of the abdomen, neurological examination and additional focused examinations if the patient’s medical history suggests presence of other disease.

***Hemoglobin, leucocytes and differential count, CRP, thrombocytes, albumin, creatinine, electrolytes, alkaline phosphatases, alanine amino transaminase. Furthermore, anti-CCP and IgM-RF are also analyzed at baseline and subsequently repeated at months 12 and 24.

****DAS28-4(crp) = 0.56*sqrt (tender joint count 28) + 0.28*sqrt (swollen joint count 28) + 0.36*ln (CRP + 1) + 0.014*GH + 0.96.

*****If technically possible.

### Medical history and demographic data

At the screening visit, information on socio-demographic data (gender, marital status, smoking history, employment and educational status), year of onset of RA symptoms, year of diagnosis, current and past medical history, comorbidity, current and past treatment with DMARDs and/or steroids and/or NSAIDS (start date, dose, frequency, end date) will be obtained, and a physical examination including height, weight, blood pressure (BP) and an electrocardiogram (ECG) will be carried out.

### Clinical and laboratory assessments

At the screening visit and at all following visits, a 40 swollen and tender joint count (right and left shoulder joints, elbow joints, wrists, MCP joints, PIP joints, 1st IP joint, knee joints, ankle joints and MTP joints) is carried out, and DAS28-CRP is calculated based on 28 joints (right and left shoulder, elbow, wrist, MCP, PIP, 1st IP and knee joints). As per standard of care, routine blood tests (hemoglobin, leucocyte and differential count, CRP, thrombocytes, albumin, creatinine, electrolytes, alkaline phosphatases and ALAT) are carried out. To ensure kidney function ahead of contrast injection in relation to MRI, a blood sample is collected for creatinine, and the glomerular filtration rate (GFR) is calculated. Blood and urine samples for biobank storage are collected at each study visit from baseline and in case the patient changes treatment between the scheduled 4-month visits.

### Patient-reported outcomes

The physical function is assessed at every visit using the Health Assessment Questionnaire (HAQ) [23]. To assess health-related quality of life, the questionnaires Short-Form 36 questionnaire (SF-36) [24,25] and EuroQol 5 dimension 5 level (EQ-5D-L5) [26] are completed at baseline and years 1 and 2.

At each visit, patient assessments of arthritis pain and fatigue and physician global assessment of disease activity on visual analog scales (0–100) and the duration of joint morning stiffness (min) will be registered.

### Imaging

MRI of unilateral 2nd to 5th MCP joints and wrist of the dominant hand will be conducted. A coronal T1-weighted 3D gradient echo sequence before and after gadolinium contrast [0.1 mmol Dotarem (gadoteric acid) per kg body weight] injection and a coronal STIR sequence before contrast injection are performed. When possible, a 2D dynamic contrast-enhanced (DCE)-MRI sequence will be performed during the first 5 min following the intravenous gadolinium contrast injection. An additional PDF file shows the acquired MRI sequences (see Additional file 1).

In the intervention group, MRI is performed at baseline and every 4 months ahead of the clinical visit. An evaluator (BE), who will be blinded to the chronology, clinical, laboratory and other imaging data, will evaluate the MR images for BME within 1 week after acquisition, and the result “BME present” or “BME absent” will be made available for the rheumatologist at the study visit. In the control group, MRI will be conducted at baseline and at years 1 and 2, but the investigator remains blinded to the results of the MRI during the study period.

Radiographs of the hands (posteroanterior view) and feet (anteroposterior view) will be conducted at baseline and yearly in both groups and will be kept blinded to the investigator.

Quality assessment of all the MRIs and radiographs will be carried out centrally by an experienced research radiographer (JM). If the MRIs or radiographs do not satisfy the quality requirements, a re-scan and/or repeat radiographs will be performed.

After study termination, separate evaluations of radiographs and MRIs will be carried out centrally. Both radiographs and MRIs will be scored with known chronology, but the readers will be blinded to clinical, laboratory and other image data. There will be two experienced readers evaluating the MRIs and x-rays, respectively.

### Electronic case report form (e-CRF)

All data registration will be recorded electronically in an e-CRF. The e-CRF has been developed to function within the nationwide DANBIO registry [27], where most Danish RA patients are already registered and followed in daily practice, making it easy to handle for the rheumatologist. The e-CRF is built of three separate communicating modules: one for the investigators, another for the radiographers and a third for the MRI evaluator. All are entering data into their module using individual usernames, passwords and user rights, i.e., the radiographers and MRI evaluators have no access to clinical data, and the MRI evaluator is blinded to patient data and only has access to anonymized imaging data. When the MRI has been evaluated, “bone marrow edema present” or “bone marrow edema absent” is entered into the CRF, and the result will then become visible to the investigator.

The e-CRF is “interactive,” i.e., only shows variables that are relevant for the visit assessed and with respect to the allocated intervention. It also specifies for the clinician when treatment should be adjusted according to the protocol. The randomization is done in the e-CRF at the baseline visit. Depending on the allocated intervention, an adjusted “order page” with a list of procedures, which have to be ordered in relation to the following visit, is available. Variables for all outcome measures assessed will be entered into the e-CRF (Table 1).

### Randomization and allocation concealment

The study utilizes a computer-generated allocation concealment process, which ensures that the group to which the patients is allocated is not known before the patient is entered into the study. Randomization and allocation are done electronically in the e-CRF at the inclusion visit. The randomization sequence is created by an independent statistician (RC) using a “random number” generator, SAS statistical software (version 9.2). The randomization sequence is entered into the e-CRF by an independent data manager (NSK). The participants will be given their study number and randomization group when the physician “clicks” on a “randomization button,” which will appear at the baseline visit. The randomization number and assigned intervention will then be visible on the screen. Thus, the patients are given consecutive screening and randomization numbers, independent of the study site.

### Blinding: outcome assessor

The IMAGINE-RA study is a randomized strategy trial. Neither patients nor the investigators are blinded to the randomization group. The evaluator evaluating the MRIs for presence/absence of BME will be blinded to the chronology, clinical, laboratory and other image data, and the investigators will be blinded to the MRIs conducted in the control group during the study period. After all visits have been completed, independent evaluators blinded to the treatment arm, clinical, laboratory and other imaging data, but not to chronology, will assess radiographs and perform full analysis of the MR images.

### Outcome measures

In Table 1 are listed the outcome measures assessed and the time points at which they are collected over the 2-year period.

### Primary outcomes

The primary *radiographic outcome* measure is presence vs. absence of radiographic progression after 2 years. Anticipating that fewer patients in the intervention group will progress on radiographs, the primary radiographic outcome measure is absence of radiographic progression from baseline to week 96, which will be assessed according to the modified Sharp/Van der Heijde score (SHS) (range 0–448) [28] separately assessing erosions and joint space narrowing in the hand, wrist and foot joints. Progression is defined as change in total SHS >0.

The primary *clinical outcome* measure is DAS28-CRP remission (DAS28-CRP <2.6) at week 96. The DAS28-CRP score, which is a composite score, will be calculated based on a joint count of 28 tender (TJC) and swollen joints (SJC) of the 40 assessed, the C-reactive protein (CRP) value and the patient’s global assessment of the disease on a visual analog scale (0–100).

### Secondary outcomes

The secondary clinical outcome measures include the presence of and changes in the following clinical findings: DAS28-CRP remission (DAS28-CRP <2.6) at week 48, ACR/EULAR remission (The 2011 ACR/EULAR Definitions of Remission in Rheumatoid Arthritis Clinical Trials) at week 48 and 96 and DAS28-CRP week 48 and 96.

The secondary imaging outcome measures include the following radiographic and MRI findings: absence of radiographic progression (Sharp/vdHeijde score) from week 0–48 and 48–96 as well as change in the Sharp/vdHeijde score from week 0–48, 0–96 and 48–96. MRI inflammatory and destructive findings are assessed using the semiquantitative OMERACT (Outcome Measures in Rheumatology) scoring system, RAMRIS (RA MRI scoring system) [29]. The following MRI secondary outcomes are assessed: absence of progression in the MRI erosion (RAMRIS) score from week 0–48 and 48–96, change in the MRI erosion (RAMRIS) score from week 0–48, 0–96 and 48–96, MRI synovitis and MRI bone marrow edema (RAMRIS) score at week 48 and 96, and change in DCE-MRI variables [including initial rate of enhancement (IRE) and maximum enhancement (ME)], if available, week 0–48 and 0–96.

Several secondary patient-reported outcomes are assessed. To assess the patients’ physical function, the Health Assessment Questionnaire Disability Index (HAQ-DI) [30] is calculated from the Health Assessment Questionnaire (HAQ). Change in HAQ-DI is calculated from week 0–48 and 0–96. To assess health-related quality of life, the questionnaires Short-Form 36 questionnaire (SF-36) [24,25] and the EuroQol 5 dimension 5 level (EQ-5D-L5) [26] are assessed at baseline and yearly. Changes in the scores are calculated from week 0–48 and 0–96.

For assessment of further secondary outcomes, blood and urine samples will be stored in a biobank for later analysis of various biomarkers.

### Statistics

#### Sample size and power considerations

Primary clinical endpoint: Assuming 60% of patients in the control group and 80% of patients in the intervention group reach the primary clinical endpoint (DAS < 2.6) at month 24, a group size of 64 in each group is needed to give a statistical power of 80% (beta = 0.20) for the detection of statistically one-sided significant (*p* = 0.05) treatment efficacy, assessed on the basis of two independent binomially distributed proportions using Pearson’s chi-square statistics with a chi-square approximation with a one-sided significance level of 0.05. Based on a specified group size of 100 patients in each group (200 in total), the project is assessed as having a 93% chance of success with regard to the clinical efficacy goal (statistical power 0.93).

Primary radiographic endpoint (assessed using the Sharp/vdHeijde score):

Assuming that 75% of patients in the control group^a^ and 90% of patients in the intervention group reach the primary radiographic performance goal (no radiographic progression) at month 24, a group size of 79 in each group is required to give a power of 80% (beta = 0.20) for detection of a statistically one-sided significant (*p* = 0.05) treatment efficacy, assessed on the basis of two independent binomially distributed proportions using Pearson’s chi-square statistics with a chi-square approximation with a one-sided significance level of 0.05. Based on the specified group size of 100 patients in each group (200 in total), the project is assessed as having an 88% chance of success with regard to the radiographic efficacy goal (statistical power 0.879).

Based on “sample size” calculations of the two above-mentioned endpoints, a total sample size of 200 has been selected.

### Statistical analysis plan

The proportion of patients in the two treatment groups who fulfill the primary endpoints will be compared using the chi-square test. This primary analysis will be based on the total intention-to-treat (ITT) population, all randomized patients independent of the adherence to the study protocol. The primary statistical analysis will be based on all usable data, irrespective of protocol deviations, and missing data will primarily be replaced using “multiple imputation” (MI), which assumes that data are ‘missing at random’ [31]. The MI analyses will be based on a “Markov chain Monte Carlo” (MCMC) simulation statement designed to fit Bayesian models to impute values for a data set with an arbitrary missing pattern, assuming a multivariate normal distribution for the data (modeled in SAS). These analyses will secondarily be supplemented by “worst-case” and “best-case” imputation in the event that data are “not missing at random” [32]. With regard to the further hypothesis-generating component of the project, a separate analysis of the patients who adhered to the study protocol (per-protocol analysis) will be carried out.

### Data registration and monitoring

The IMAGINE-RA study is carried out in accordance with the approved protocol and the applicable regulatory requirements and legislation in this field. A good clinical practice (GCP) monitoring plan has been developed in cooperation with the GCP unit in Copenhagen to ensure that the trial is carried out, registered and reported in accordance with the protocol, with specified procedures and with Danish law. Two internal monitors who will ensure that all data have been entered correctly and in accordance with the protocol will carry out monitoring. If data are missing, queries will be sent electronically to the sites. All study information will be recorded in the e-CRF, which is developed to function within the Danish DANBIO registry. The e-CRFs will be completed by the investigator, project nurse and patient. To get access to the e-CRF, a username and password are required.

### Ethics approval

The regional Scientific Ethical Committee of Southern Denmark has approved the study (project ID: S-20110109). The study is carried out according to the Declaration of Helsinki 2008 (*Declaration of Helsinki* World Medical Association 2008) fulfilling all general ethical considerations. All participants will receive oral and written information about the study, and oral and written informed consent is obtained, with the possibility to drop out at any time.

### Storage of personal information/Danish data protection agency

To give the health authorities the option of further evaluation, the investigator registers, *inter alia*, the identities of all participating patients (enough information to link the registrations, e.g., CRFs and hospital journals) and stores all original signed informed consent forms and electronic CRFs. In accordance with international regulations, the investigator stores these registrations for a minimum of 10 years. Patient data are protected in accordance with the Danish Act on the Processing of Personal Data and the Danish Health Act. The Danish Data Protection Agency has approved the project.

### Adverse events

It is the investigator’s responsibility to ensure that all serious adverse reactions/adverse events are immediately reported to the sponsor and project leader, who are responsible for notifying the regional Biomedical Research Ethics Committee. Reports to the regional Biomedical Research Ethics Committee must be accompanied by comments on the possible consequences for the trial. The project leader is also responsible for informing the participating departments of what a serious adverse reaction/adverse event entails. It is also the responsibility of the project leader to submit on an annual basis, starting 1 year after approval of the study, a list of all serious unexpected adverse reactions and serious adverse events that have occurred in the period to the regional Biomedical Research Ethics Committee. Reports must be accompanied by an assessment of the safety of trial subjects.

The study was designed by the authors. Input from AbbVie was received only at the onset of the protocol writing. Data will be collected by the monitoring company (ZiteLab) and will be assessed jointly by the senior biostatistician (RC) and the group authors, without interference from the sponsor. The data will be interpreted and the report written by the authors without writing support from the sponsor. The corresponding author will have full access to all the data in the study and have the final responsibility for the decision to submit for publication.

## Discussion

This study is expected to contribute significant new knowledge about the options for improved outcome for RA patients by better and more sensitive disease monitoring using MRI and thereby ensuring optimal clinical and radiographic disease control. The use of MRI as a treatment guide may be valuable in individualizing the treatment, so patients assessed as being at high risk of erosive progression can receive appropriately intensive treatment so disease progression can be avoided. If an MRI-guided treatment strategy is shown to be beneficial, implementation in daily clinical practice will also be feasible. The availability of MRI can vary, but the examination time doing a scan for BME is short (less than 30 min), and no contrast agent has to be administered. Thus, the procedure will be noninvasive and safe for the patient. In addition, the MRIs only need to be evaluated for the presence or absence of BME, meaning that the evaluator will not need to be trained in a specific scoring system.

The design of this study is unique and methodologically very strict and systematic, which is essential to achieve scientifically valid data. A weakness is however the non-blinded design. The rheumatologist and patient are not blinded to the allocated intervention, which potentially could lead to bias. However, full blinding would require that all seven MRIs also were done in the control group, but not used, which was not considered feasible and ethically justified. A major strength is that all the MRI scans will be evaluated for BME within 7 working days after acquisition at a central reading center by one evaluator (BE), who has extensive experience from previous MRI studies [10,19,33,34]. The evaluator will be blinded to chronology, clinical, laboratory and other image data. Consequently, the evaluations will be consistent, which will improve study quality and the subsequent results.

The IMAGINE-RA trial is, as far as we are aware, the first study using MRI to guide RA treatment. The results of the study will be of great importance for all rheumatologists that treat RA patients and can potentially change our way of monitoring RA patients and individualizing the treatment so a better disease outcome can be achieved.

## Trial status

The trial is recruiting.

## Endnotes

^a^The AMBRA study at King Christian 10th Hospital for Rheumatic Diseases, Graasten and Vejle Hospital, involved 300 patients with RA in accordance with the ACR 1987 classification criteria, DAS28 < 3.2, ≥1½ year’s disease duration, unchanged anti-rheumatic treatment ≥ 3 months and no biological treatment. The patients were monitored in different outpatient courses, treated with DMARDs and where necessary given biological treatment to maintain low disease activity based on clinical evaluation. Radiographic progression in the wrists, hands and feet 0–24 months was seen in 25% of patients who were anti-CCP positive and had erosive disease at the time of inclusion. The proportion of patients with DAS28 > 2.6 was 38% [23].

## Additional file

Additional file 1:
**Acquired MRI-sequences in the IMAGINE-RA study.** Description of acquired MRI sequences of the wrist and MCP joints in the IMAGINE-RA study.

## Abbreviations

ACR: American college of rheumatology
Anti-CCP: Anti-citrullinated peptide antibodies
BME: Bone marrow edema
DAS: Disease activity score
DCE-MRI: Dynamic contrast-enhanced MRI
DMARDs: Disease-modifying anti-rheumatic drugs
ESR: Erythrocyte sedimentation rate
EQ-5D: EuroQol 5 dimension 5 levels
EULAR: European league against rheumatism
HAQ: Health assessment questionnaire
HAQ-DI: Health assessment questionnaire disability index
ITT: Intention to treat
MRI: Magnetic resonance imaging
RA: Rheumatoid arthritis
RAMRIS: RA MRI scoring system
RCT: Randomized controlled trial
SF-36: Short-form 36 questionnaire
