# Supplementary material for: Impact of a magnetic resonance imaging-guided treat-to-target strategy on disease activity and progression in patients with rheumatoid arthritis (the IMAGINE-RA trial): study protocol for a randomized controlled trial
Source: Trials. 2015 Apr 21;16:178. doi: 10.1186/s13063-015-0693-2 (PMC4417239; doi:10.1186/s13063-015-0693-2)
Supplement: Additional file 1: — Acquired MRI-sequences in the IMAGINE-RA study. Description of acquired MRI sequences of the wrist and MCP joints in the IMAGINE-RA study. [file 13063_2015_693_MOESM1_ESM.pdf]

Details of acquired MRI sequences in the IMAGINE-RA study

MRI of the 2nd to 5th MCP joints and wrist joint is performed at the same time, if the FOV is large enough to cover both regions (Table 1). Alternatively separate scans of MCP and wrist joints using individual FOVs are performed (Table 2).

Table 1: Applied MRI sequences, when MCP joints and wrist joint are covered by the same FOV

No.	Type	Plane	Region	ST (mm)	Gap (mm)	Resolution (mm)*	No. slices	FOV (mm)	Averages	Time (min)
1	Localizer	Ax, cor, sag	Wrist + MCP							
2	STIR	Cor	Wrist + MCP	2.5	0.6	0.6x0.6	15	180x145	4	App. 5
3	T1 FFE 3D	Cor	Wrist + MCP	0.8 (0.4)	-	0.8x0.8 (0.4x0.4)	77	180x150	1	App. 5
4	T1 FFE 3D (dyn)	Cor	Wrist + MCP	2 (1)	-	1.5x1.5 (0.75x0.75)	20	180x80	1	6.5 sec. (repeated for 4 minutes)
5	T1 FFE 3D	Cor	Wrist + MCP	0.8 (0.4)	-	0.8x0.8 (0.4x0.4)	77	180x150	1	App. 5

Table 2: Applied MRI sequences, when MCP joints and wrist joint are scanned separately.

No	Type	Plane	Region	ST (mm)	Gap	Resolution (mm)*	No. slices	FOV (mm)	Averages	Time (min)
	Positioning A		MCP							
1	Localizer	Ax, cor, sag	MCP							
2	STIR	Cor	MCP	2.5	0.6	0.6x0.6	15	180x145	4	App. 5
3	T1 FFE 3D	Cor	MCP	0.8 (0.4)	-	0.8x0.8 (0.4x0.4)	77	180x150	1	App. 5
	Positioning B		Wrist							
4	Localizer	Ax, cor, sag	Wrist							
5	STIR	Cor	Wrist	2.5	0.6	0.6x0.6	15	180x145	4	App. 5
6	T1 FFE 3D	Cor	Wrist	0.8 (0.4)	-	0.8x0.8 (0.4x0.4)	77	180x150	1	App. 5
	3D dyn		Wrist							
i.v Dotarem (gadoteric acid) 0.2 ml/kg as bolus										
7	T1 FFE 3D (dyn)	Cor	Wrist	2 (1)	-	1.5x1.5 (0.75x0.75)	20	180x180	1	6.5 sec. (repeated for 4 minutes)
8	T1 FFE 3D	Cor	Wrist	0.8 (0.4)	-	0.8x0.8 (0.4x0.4)	77	180x150	1	App. 5
	Positioning A		MCP							
9	Localizer	Ax, cor, sag	MCP							
10	T1 FFE 3D	Cor	MCP	0.8 (0.4)	-	0.8x0.8 (0.4x0.4)	77	180 x 150	1	App. 5

* When 2 sets of values are provided, e.g. ”0.8x0.8 (0.4x0.4)”, images are acquired with the first-mentioned slice thickness (e.g. 0.8 mm) but are reconstructed in the last-mentioned slice thickness (e.g. 0.4 mm).

Ax: Axial; Cor: Coronal; Dyn: Dynamic; FFE: Fast Field Echo; FOV: Field of view; I.v: Intravenous; MCP: Metacarpophalangeal joint; Sag: Sagittal; ST: Slice thickness; STIR: Short Tau Inversion Recovery; 3D: 3 Dimensional
